# Supplementary figures and images for: A Network-Based Approach for Identification of Subtype-Specific Master Regulators in Pancreatic Ductal Adenocarcinoma
Source: Genes (Basel). 2020 Feb 1;11(2):155. doi: 10.3390/genes11020155 (PMC7074188; doi:10.3390/genes11020155)

a

**miR-29c regulon**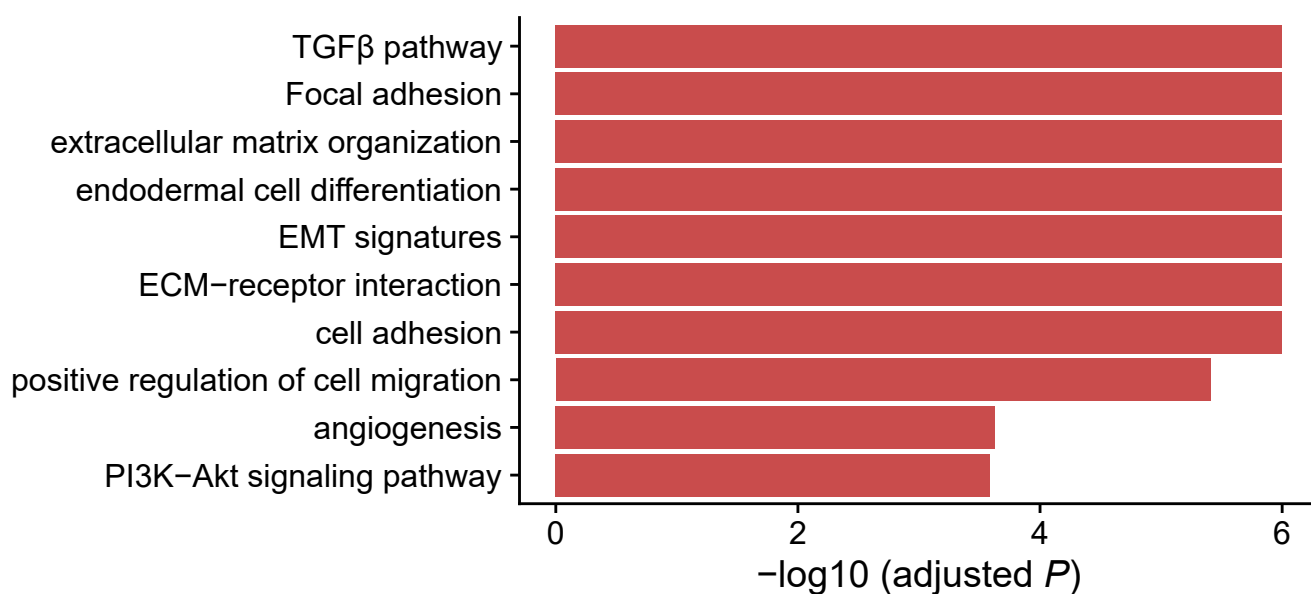

b

**miR-192 regulon**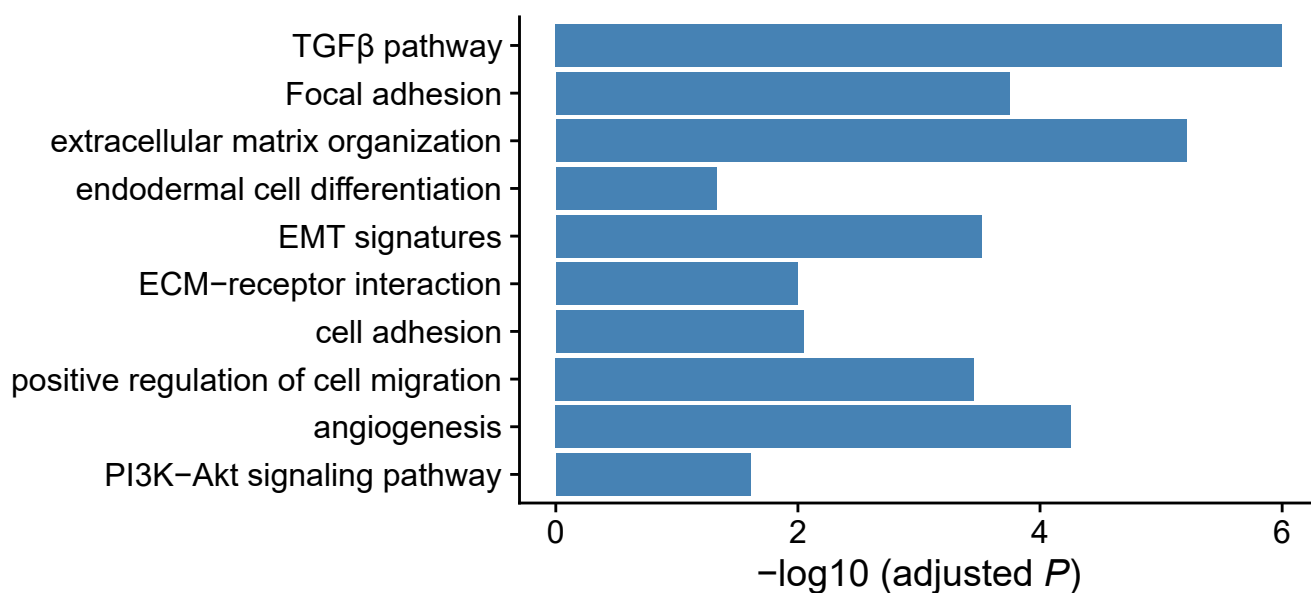

Supplement: Supplementary file 1 [file genes-11-00155-s001.zip › Supplementary_20200127/Figure S1.pdf]

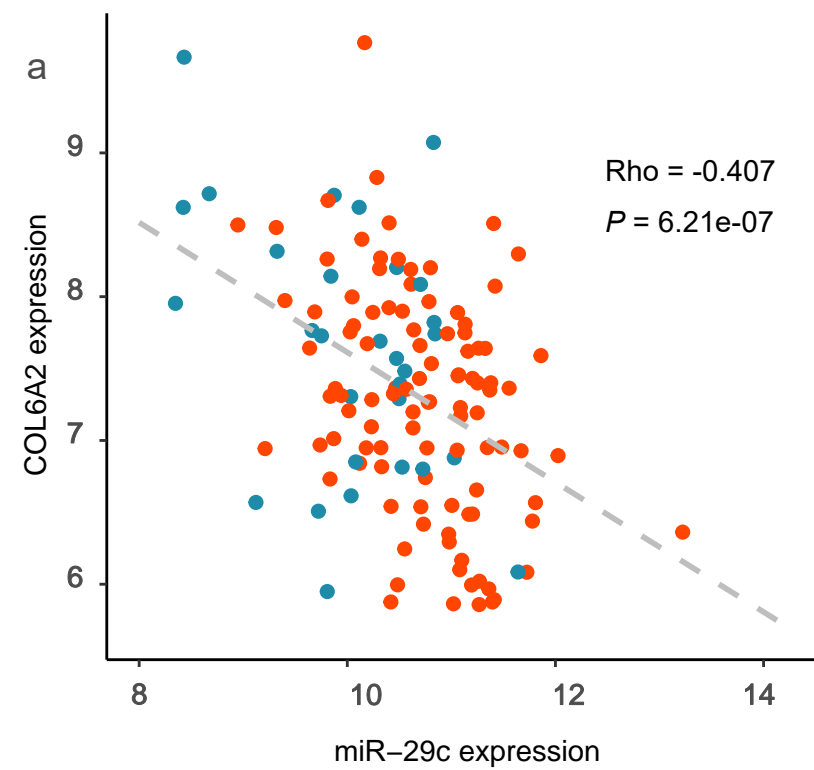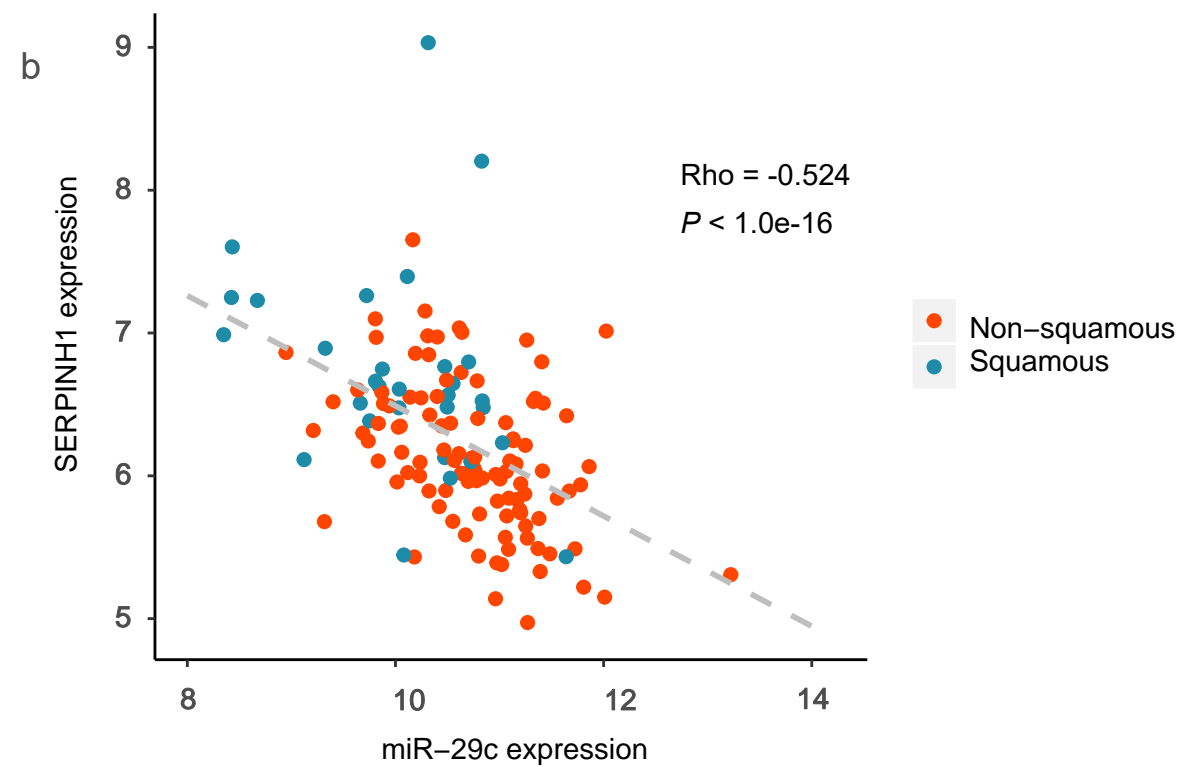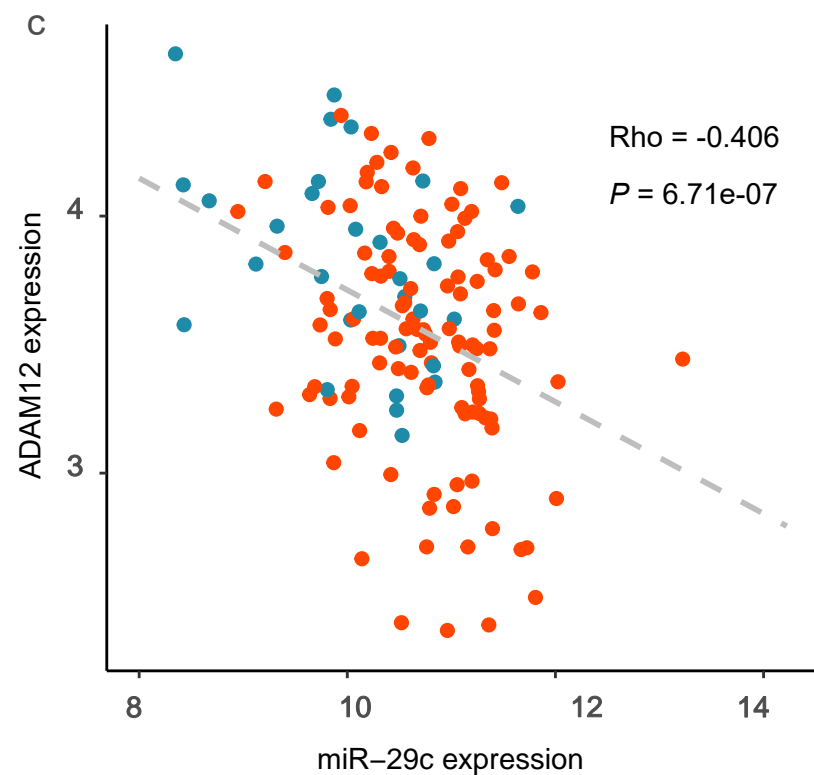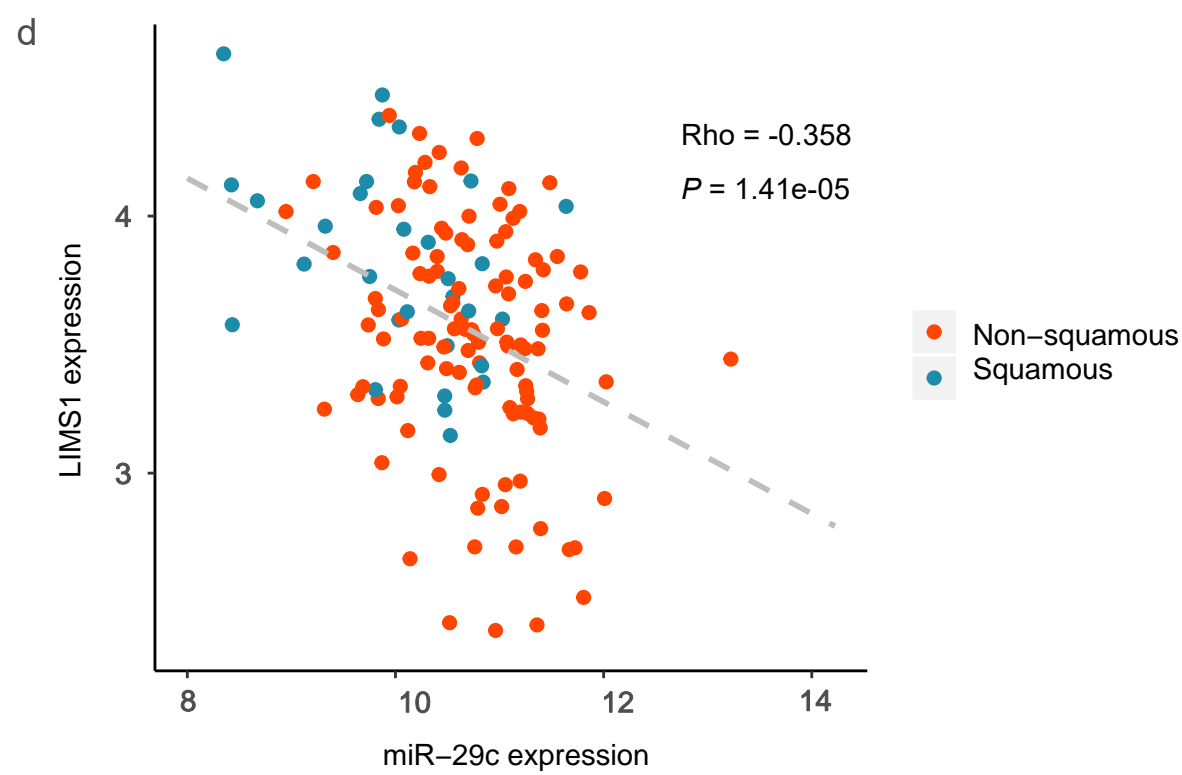

Supplement: Supplementary file 1 [file genes-11-00155-s001.zip › Supplementary_20200127/Figure S2.pdf]

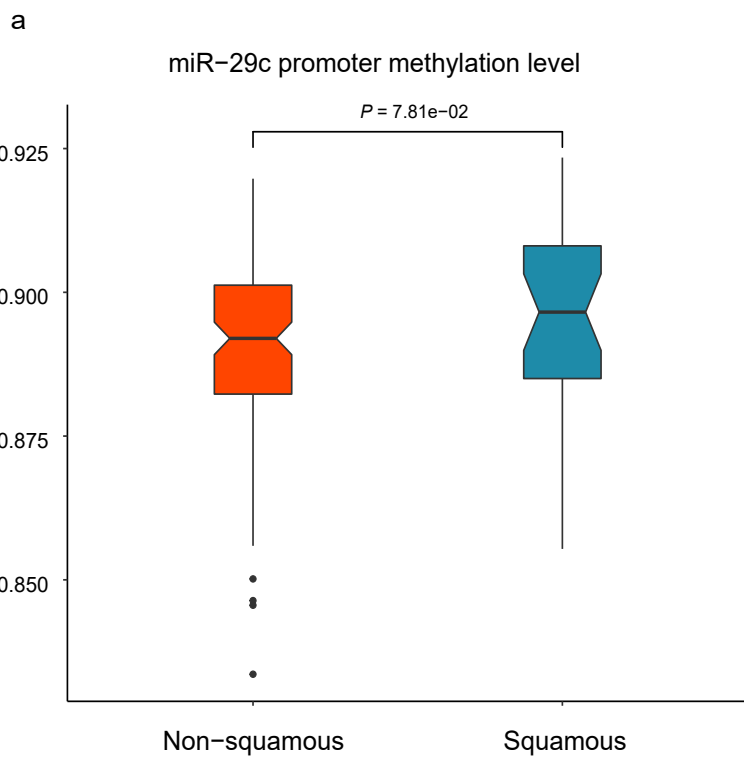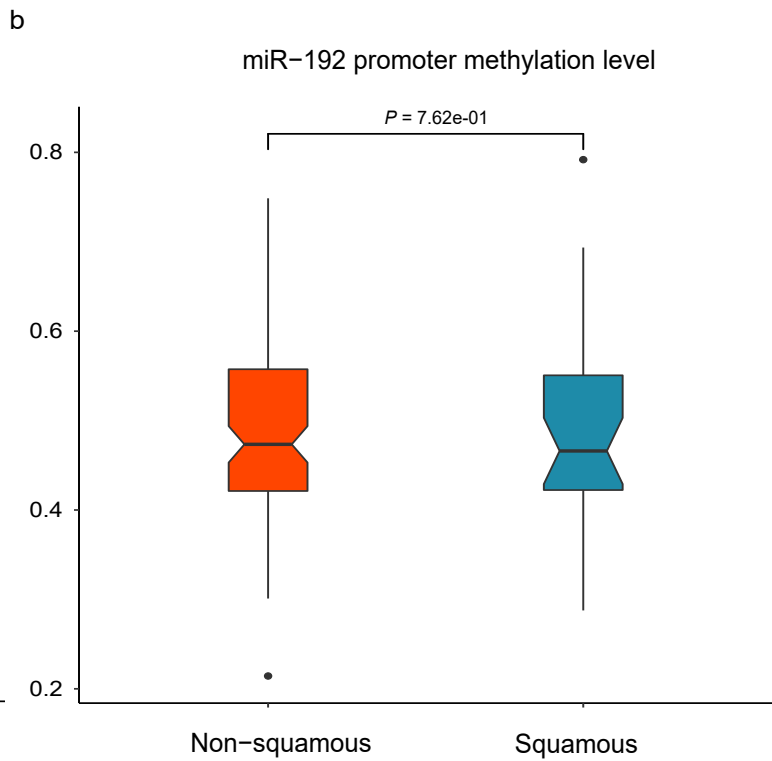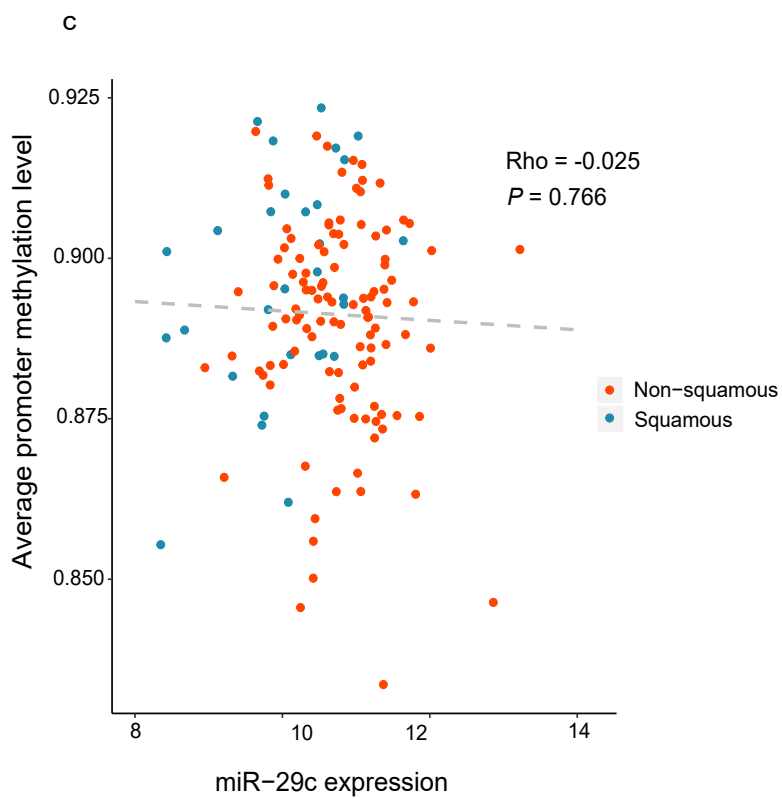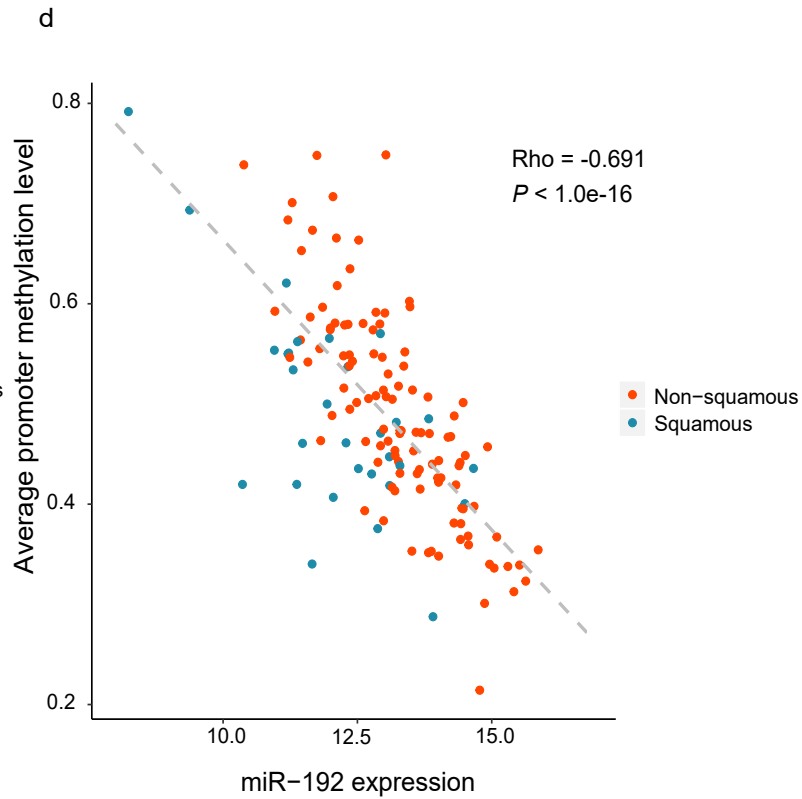

Supplement: Supplementary file 1 [file genes-11-00155-s001.zip › Supplementary_20200127/Figure S3.pdf]

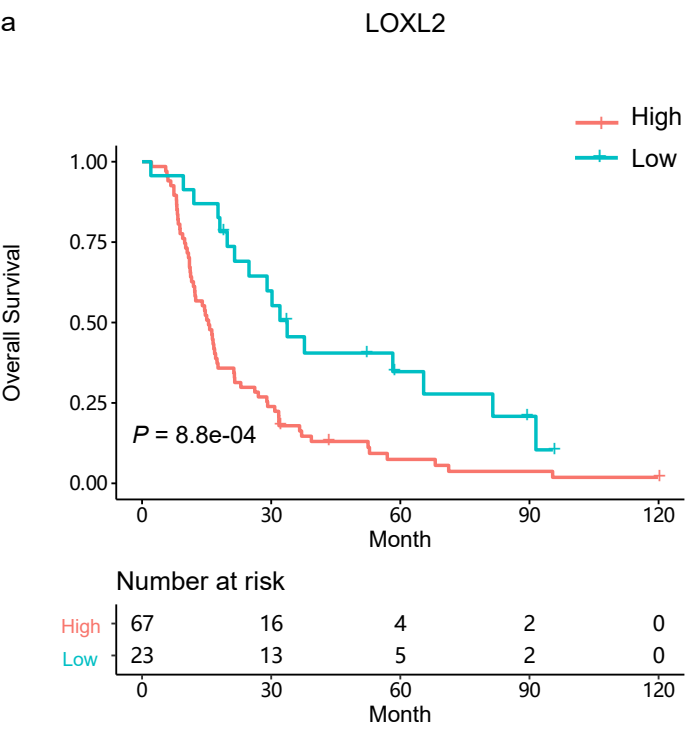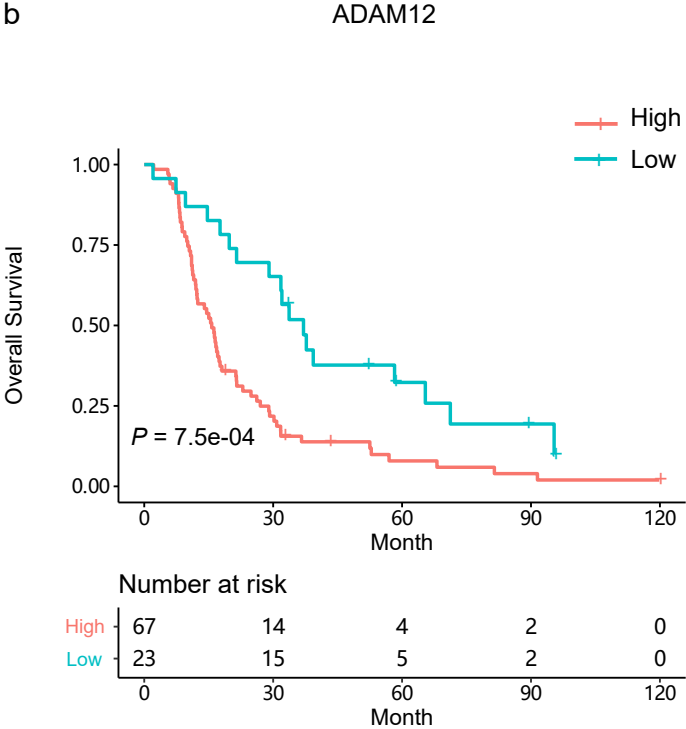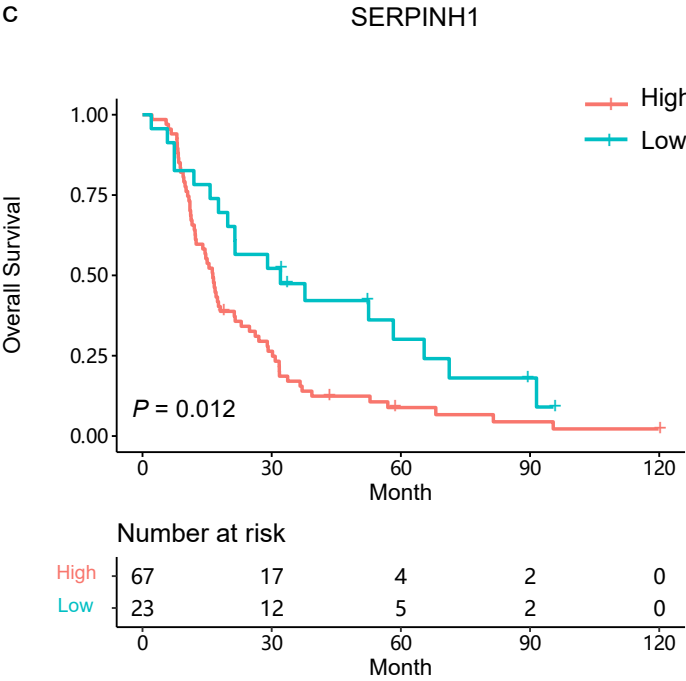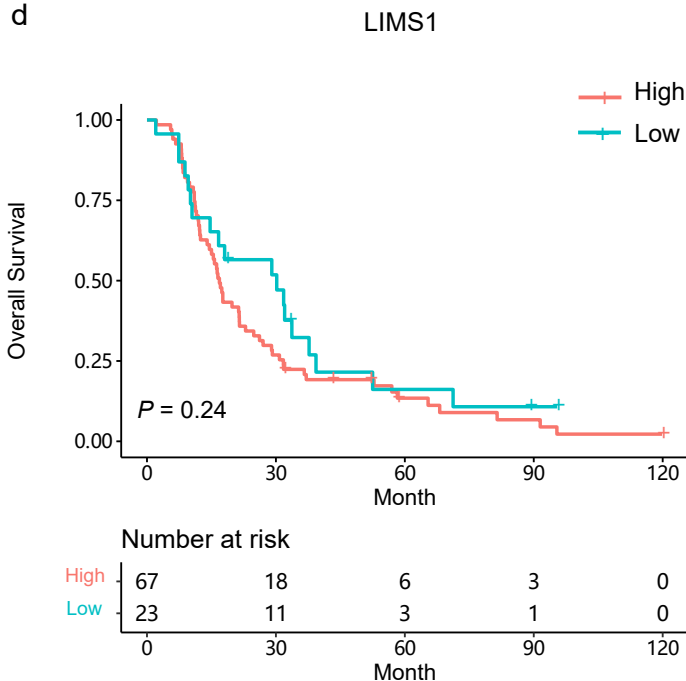

Supplement: Supplementary file 1 [file genes-11-00155-s001.zip › Supplementary_20200127/Figure S4.pdf]
